# Single-Layer High-Efficiency Metasurface for Multi-User Signal Enhancement

**DOI:** 10.3390/mi16080911

**Published:** 2025-08-06

**Authors:** Hui Jin, Peixuan Zhu, Rongrong Zhu, Bo Yang, Siqi Zhang, Huan Lu

**Affiliations:** 1 State Key Laboratory of Extreme Photonics and Instrumentation, ZJU-Hangzhou Global Scientific and Technological Innovation Center, Zhejiang University, Hangzhou 310027, China; 13706818052@139.com (H.J.); peixuanzhu@zju.edu.cn (P.Z.); sjgqzhang@outlook.com (S.Z.); 2 Zhejiang Key Laboratory of Intelligent Electromagnetic Control and Advanced Electronic Integration, Jinhua Institute of Zhejiang University, Zhejiang University, Jinhua 321099, China; 3 International Joint Innovation Center, The Electromagnetics Academy at Zhejiang University, Zhejiang University, Haining 314400, China; 4 School of Information and Electrical Engineering, Hangzhou City University, Hangzhou 310015, China; 5 Sussex Artificial Intelligence Institute, Zhejiang Gongshang University, Hangzhou 310018, China; 18867645958@163.com

**Keywords:** high efficiency, metasurface, signal enhancement

## Abstract

In multi-user wireless communication scenarios, signal degradation caused by channel fading and co-channel interference restricts system capacity, while traditional enhancement schemes face challenges of high coordination complexity and hardware integration. This paper proposes an electromagnetic focusing method using a single-layer transmissive passive metasurface. A high-efficiency metasurface array is fabricated based on PCB technology, which utilizes subwavelength units for wide-range phase modulation to construct a multi-user energy convergence model in the WiFi band. By optimizing phase gradients through the geometric phase principle, the metasurface achieves collaborative wavefront manipulation for multiple target regions with high transmission efficiency, reducing system complexity compared to traditional multi-layer structures. Measurements in a microwave anechoic chamber and tests in an office environment demonstrate that the metasurface can simultaneously create signal enhancement zones for multiple users, featuring stable focusing capability and environmental adaptability. This lightweight design facilitates deployment in dense networks, providing an effective solution for signal optimization in indoor distributed systems and IoT communications.

## 1. Introduction

In the realm of modern wireless communications, maintaining signal quality in high-density multi-user scenarios stands as a pivotal challenge impeding the enhancement of system capacity [1,2,3,4]. With the exponential growth of Internet of Things (IoT) devices and the widespread deployment of WiFi 6/6E standards, indoor wireless environments are increasingly characterized by severe multipath fading, dense co-channel interference, and uneven coverage—particularly in crowded spaces such as offices, shopping malls, and residential complexes [5,6,7]. These issues are exacerbated in the 5 GHz WiFi band, where higher propagation loss and susceptibility to obstacles further degrade signal integrity, leading to reduced data rates, increased latency, and unreliable connections for end-users [8,9,10].

Traditional signal enhancement approaches have struggled to address these challenges effectively. Macro-cell densification and relay insertion introduce high hardware costs and complex coordination between nodes, while fully digital beamforming relies on expensive radio-frequency (RF) chains and power-hungry signal processing modules, making them impractical for large-scale indoor deployment [11,12]. Reconfigurable intelligent surfaces (RISs) have emerged as a promising alternative, but active RIS designs often suffer from narrow bandwidths, high insertion loss, and cumbersome integration with existing WiFi infrastructure [13,14,15].

Metasurfaces, as subwavelength artificial electromagnetic (EM) structures, offer a revolutionary paradigm for wavefront manipulation through their ability to independently modulate the phase, amplitude, and polarization of EM waves [16,17,18,19]. Recent advances in transmissive metasurfaces have demonstrated potential for signal focusing, but critical limitations remain: Multi-layer designs, while achieving wide phase coverage, suffer from significant interlayer reflection losses and narrow operational bandwidths, failing to span the 5.2–5.8 GHz WiFi band [20,21,22,23,24]. Single-layer structures, on the other hand, often compromise phase coverage range for efficiency, or rely on complex resonant mechanisms that restrict multi-target focusing capability [25,26,27,28,29]. In multi-user scenarios, these limitations result in uneven signal enhancement, poor environmental adaptability, and high deployment complexity—hindering their practical adoption in indoor networks [30,31,32].

Addressing these challenges, this study introduces a single-layer transmissive metasurface designed for operation in the WiFi frequency band (5.2–5.7 GHz) [33,34,35,36]. By leveraging geometric phase principles to optimize subwavelength unit cell design, the proposed structure achieves a 300° wide-range phase coverage and a transmission efficiency exceeding −2 dB, thereby overcoming the efficiency and bandwidth constraints of conventional multi-layer configurations [37,38,39,40]. An array system constructed from this metasurface demonstrates dual-position EM focusing in a microwave anechoic chamber and enables the creation of signal enhancement zones for multiple user terminals in real-world office environments, validating its stable focusing performance and engineering feasibility in complex scenarios [41,42,43]. This lightweight, low-power solution paves the way for the practical implementation of metasurface technologies in indoor distributed networks and IoT communications, marking a significant stride toward their broader adoption in wireless communication systems [44,45].

## 2. Results

**Figure 1:**
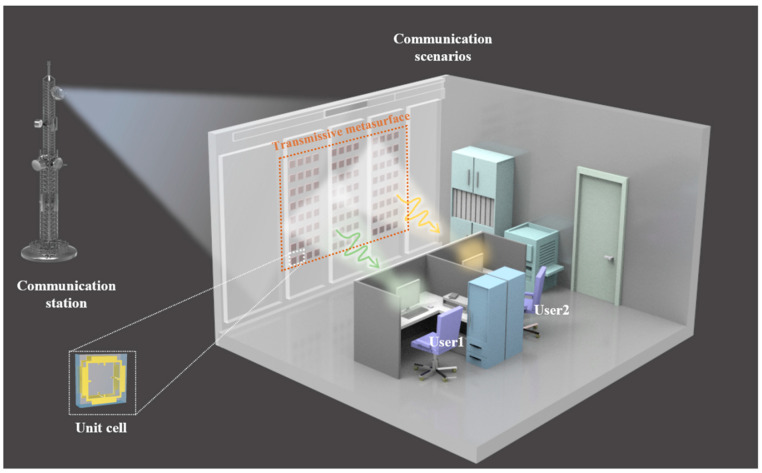
The proposed multi-user communication scenario.

Figure 1 depicts a schematic diagram of a dual-user communication scenario. The communication base station, as the core infrastructure of the communication network, undertakes the function of transmitting and receiving wireless signals, and realizes network coverage of the target area by establishing wireless communication links with terminal devices (such as indoor user terminals). In the proposed office communication scenario constructed on the right side of the figure, the transmissive metasurface array marked by orange dashed lines is designed based on subwavelength unit structures, and the green and orange arrows respectively characterize the wireless transmission paths between the base station and different users. The transmissive metasurface ensures the reliable transmission of information from the transmitter to the receiver by regulating the amplitude and phase characteristics of EM waves, focuses the signal energy on the specified area, and achieves signal enhancement in dual-user and multi-user scenarios.

**Figure 2:**
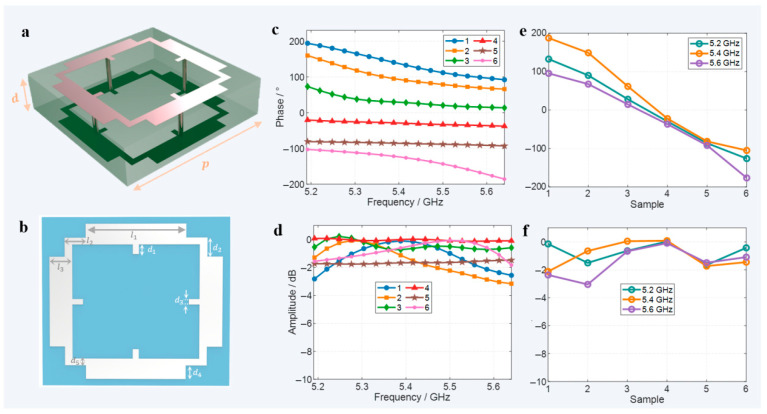
Unit cell and EM responses. (**a**) Schematic of the overall unit cell structure. (**b**) Top view of the unit cell structure. (**c**) Transmission phase responses of the unit cell at different frequencies, with six representative curves selected. (**d**) Amplitude responses of the unit cell at different frequencies. (**e**) Phase coverage of six representative unit cells at the operating frequency points. (**f**) Transmission amplitudes of six representative unit cells at the operating frequency points.

To realize the communication scenario shown in Figure 1, the design of unit cells with high transmittance and wide phase coverage is initially required. Figure 2 illustrates the broadband transmissive unit cell structure and its EM response characteristics. Figure 2a shows the schematic of the overall unit cell structure, which adopts a single-layer dielectric design with symmetric metal layers on the top and bottom surfaces. Electrical connections are realized through metallized vias around the structure, with a thickness of d = 5 mm and a unit cell side length of *p* = 20 mm. Figure 2b presents the top view of the unit cell structure, where l1,  l2,  l3,  d1,  d2,  d3,  d4,  and d5 are all adjustable structural parameters. Differentiated phase and amplitude response characteristics can be achieved through different parameter combinations.

The core mechanism of this design can be explained by the correlation between EM theory and structural characteristics. The unit adopts a substrate with a relative permittivity ε_r_ = 2.65 (thickness 5 mm and length 20 mm). From the perspective of impedance matching, the reflection coefficient at the air–substrate interface under normal incidence is calculated according to the Fresnel formula: Γ = (η_2_ − η_1_)/(η_2_ + η_1_), where η_0_ = 377 Ω is the free-space wave impedance, and the substrate wave impedance η_2_ = η_0_/√ε_r_ ≈ 228 Ω. Substituting the values, we get Γ ≈ −0.22, which lays the foundation for high transmittance. The 5 mm substrate thickness is approximately 1/10–1/11 of the operating wavelength λ_0_ (53.5–57.7 mm), which can suppress multimode interference in the substrate. Combined with the symmetric metal patterns on the top and bottom layers, mode conversion loss is further reduced. The 20 mm sub-wavelength size avoids high-order diffraction between units, ensuring stable array performance.

In terms of phase control, basic adjustment is achieved through the geometric phase principle. The four metal vias connect the top and bottom metal layers to form a three-dimensional resonant structure: the LC circuit formed by the vias and metal patterns (resonant frequency f_0_ = 1/(2π√(LC)), after parameter optimization, has a resonant frequency band covering 5.2–5.6 GHz. Combined with the enhanced coupling effect of the three-dimensional current path, a continuous phase coverage of over 300° is finally achieved. This design not only retains the advantage of the single-layer structure in reducing interlayer reflection (reducing 2–3 interface losses compared with the multi-layer structure) but also expands control freedom through vertical currents, balancing transmittance and phase continuity in the wide frequency band.

Six typical unit cell structures were selected for EM response testing, and their transmission phase and amplitude characteristics in the operating frequency band (5.2–5.6 GHz) are shown in Figure 2c–f. Figure 2c gives the transmission phase curves of the six unit cell structures at different frequencies, demonstrating that the phase coverage range can reach approximately 300°. Figure 2d shows the corresponding amplitude response curves, with transmission amplitudes all better than −3 dB and the highest reaching −0.02 dB. Further selecting 5.2 GHz, 5.4 GHz, and 5.6 GHz as representative operating frequency points, Figure 2e displays the phase coverage ranges of the six unit cells at these three frequency points, which are 270°, 300°, and 290°, respectively. Figure 2f shows the transmission amplitude characteristic curves at the operating frequency points. With the exception of the amplitude of the second unit cell that is −3 dB at 5.6 GHz, the amplitudes of the remaining units are all controlled within −2 dB. The wide phase coverage range and high transmission efficiency of this structure provide important support for realizing the multi-user focusing function. Supplementary Information S1, S2, and S3 analyze the electromagnetic responses of the unit structure under different substrate thicknesses, different incident angles, and flexible substrates, respectively.

In Table 1, we compare our work (taking the central frequency of 5.4 GHz as an example) with reported microwave achromatic lenses in terms of the number of structural layers, applicability in the WiFi frequency band, and efficiency. It can be seen that the efficiency of our design is superior to that of other works.

**Figure 3:**
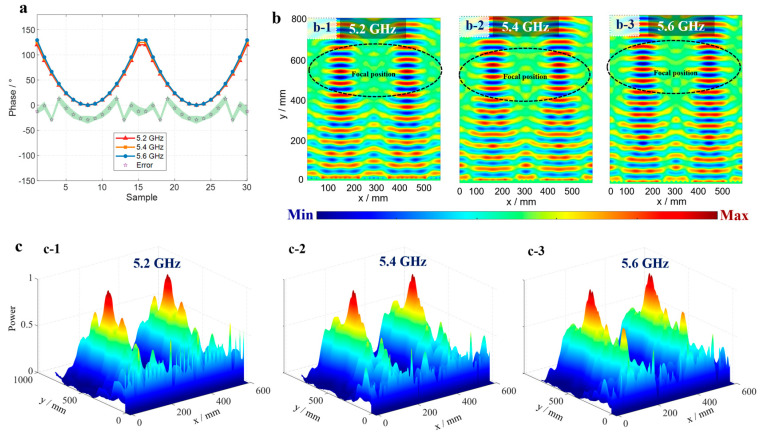
Simulation results of dual-point focusing. (**a**) Theoretical focusing phases and error phases. (**b**) Real-part results of dual-point focusing at different frequencies. (**c**) Energy diagrams of dual-position focusing at different frequencies.

Based on the above-mentioned unit cell structure, a metasurface array with the scale of 600 mm × 400 mm was constructed. According to EM theory, the phase that the metasurface needs to compensate for realizing dual-position focusing can be expressed as follows:

(1) $$\phi (x)=\frac{2\pi }{\lambda }[\sqrt{{\left(x-x_{1}\right)}^{2}+y_{1}^{2}}+\sqrt{{\left(x-x_{2}\right)}^{2}+y_{2}^{2}}-(y_{1}+y_{2})]$$

where $\phi \left(x\right)$ represents the phase modulation value to be applied at the coordinate x of the metasurface. $y_{1}$ and $y_{2}$ denote the vertical distances from the focal points to the metasurface (in the *y*-axis direction), respectively. λ represents the operating wavelength of the incident EM wave. ($x_{1},y_{1}$) is the two-dimensional coordinate of the first target focal point, where $y_{1}$ is the vertical distance from the focal point to the metasurface. ($x_{2},y_{2}$) is the two-dimensional coordinate of the second target focal point, where $y_{2}$ is the vertical distance from the focal point to the metasurface. The energy-focusing positions are set as (150 mm, 500 mm) and (450 mm, 500 mm), respectively. The theoretical compensation phases of each metasurface unit at the operating frequency points are calculated by Formula (1) as shown in Figure 3a. The red, orange, and blue solid lines in the figure correspond to the phase compensation curves at 5.2 GHz, 5.4 GHz, and 5.6 GHz, respectively. Due to the close operating frequencies, the three curves show good consistency. The star-shaped scatter points in the figure are the error distributions between the actual calculated phases and the theoretical values, and the green shaded area represents the confidence interval of the error fluctuation within ±10°.

Figure 3b shows the real-part distributions of the electric field for dual-position focusing at different frequencies, where the abscissa is the size of the metasurface, and the ordinate is the focal length parameter. Subfigures b-1, b-2, and b-3 correspond to the simulation results at 5.2 GHz, 5.4 GHz, and 5.6 GHz, respectively. It can be seen that after being regulated by the metasurface, the real-part energy of the electric field is concentrated at the theoretical focusing positions marked by black dashed lines. To further quantify focusing performance, electric field energy distributions at different frequencies are simultaneously plotted in Figure 3c. Subfigures c-1, c-2, and c-3 correspond to the results at the three operating frequency points (the s-axis is the metasurface size; the *y*-axis is the focal length; and the vertical axis characterizes the energy intensity). Through calculation, the actual focal positions (position 1) at each frequency are (160 mm, 520 mm), (148 mm, 530 mm), and (165 mm, 515 mm), respectively; the focal positions (position 2) at each frequency are (490 mm, 515 mm), (468 mm, 525 mm), and (480 mm, 520 mm), respectively, which are in good agreement with the theoretical focusing positions.

Based on the characteristics of subwavelength structures, PCB technology is used for plate manufacturing. An F4B plate with a relative permittivity of 2.65 is selected, and the thickness of metallic copper on the plate surface is 18 microns. The metasurface coating is transferred onto the metal foil using an etching process. A photoresist is applied to the metal foil, which is then exposed through a photomask and developed to reveal the metal patterns that need to be retained; a chemical etching solution (such as ferric chloride) is used to corrode the metal not protected by the photoresist, ultimately leaving the metal unit structures consistent with the design. The dielectric layers covered with metal patterns are aligned and pressed under high temperature (150–200 °C) and high pressure (10–30 MPa) to cure and bond the dielectric layers, forming an integral substrate.

Metallized vias are conductive channels connecting metals of different layers in the PCB, and they are used to electrically connect the upper and lower metal layers in the metasurface. Their fabrication is a key step in the PCB process. A precision drilling machine (or laser drilling machine) is used to drill holes at designed positions on the PCB substrate. A thin layer of copper (approximately 0.5–1 μm) is deposited on the hole walls through electroless copper plating (electroless plating) to form a conductive seed layer; the copper layer inside the holes is thickened to 20–50 μm through electrolytic plating to ensure good conductivity of the vias and reliable connection with the upper and lower metal foils (forming an integral conductive structure). Finally, the designed metasurface array is processed and formed.

**Figure 4:**
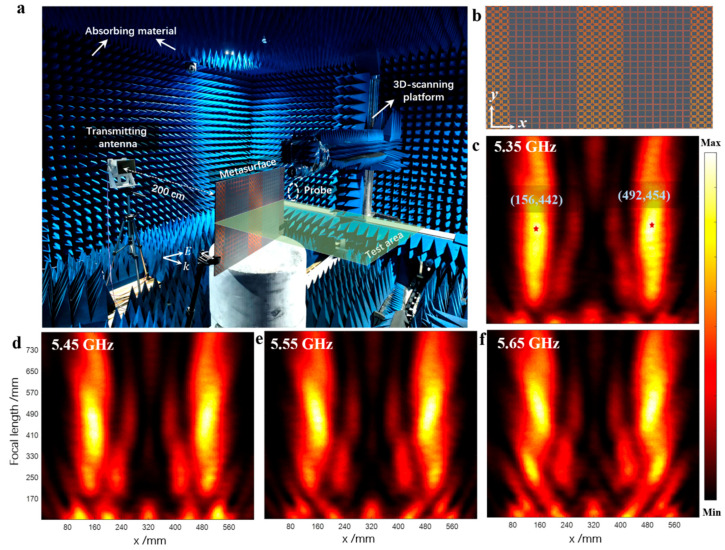
Experimental results in microwave anechoic chamber. (**a**) Experimental setup. (**b**) Fabricated metasurface device. (**c**–**f**) Focusing effects of the metasurface at different frequencies.

To verify dual-position focusing performance, a test environment as shown in Figure 4a was established. The transmitting antenna was placed approximately 200 cm away from the metasurface to ensure that the incident EM wave approximated a plane wave. The metasurface was mounted at a designated position on a three-dimensional scanning device, and a probe was used as the receiving antenna, fixed in the central area of the metasurface to collect electric field data. The green detection area was set as a rectangular range of 600 mm × 750 mm. Figure 4b shows the fabricated metasurface array, which contains 30 unit cells in the x-direction and 20 unit cells in the periodic y-direction.

The test results of electric field energy distributions at different frequencies are shown in Figure 4c–f (with the abscissa as the metasurface size and the ordinate as the focal length). The focusing results at 5.35 GHz are shown in Figure 4c, where the energy peak positions (marked by red stars) are (156 mm, 442 mm) and (492 mm, 454 mm), respectively. The electric field distribution at 5.45 GHz is shown in Figure 4d, with corresponding focusing positions at (160 mm, 485 mm) and (480 mm, 475 mm). The measured focal positions at 5.55 GHz and 5.65 GHz are (160 mm, 460 mm), (480 mm, 470 mm), (160 mm, 490 mm), and (480 mm, 492 mm), respectively (see Figure 4e,f). Although there are certain deviations between the measured results and the theoretical calculations/simulation data (possibly caused by processing errors and phase discretization errors), the energy can generally focus near the theoretical positions, successfully achieving the dual-focus distribution effect.

**Figure 5:**
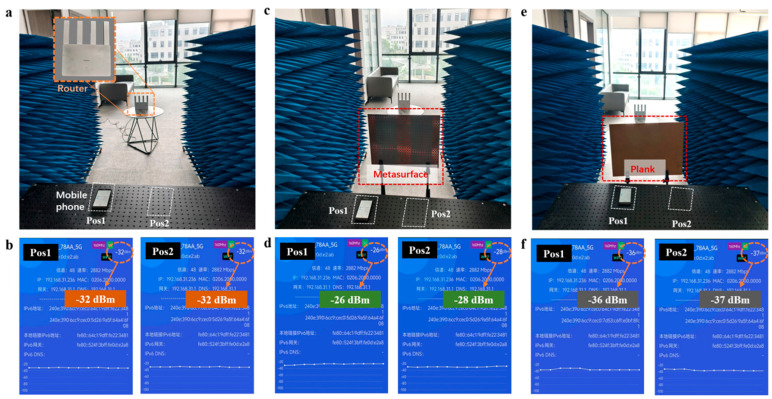
Signal enhancement tests in office environment. (**a**) Test environment without obstruction and metasurface. (**b**) Test environment with metasurface placed between router and mobile phone. (**c**) Test environment with obstruction between router and mobile phone. (**d**) The signal strength received by users at different positions. (**e**) Test environment with obstruction between router and mobile phone. (**f**) The signal strength received by users at different positions.

Figure 5 demonstrates comparative test results of metasurface-enhanced WiFi signal in office environments. The experiment utilized smartphones as signal detection terminals, verifying focusing performance through real-time monitoring of signal strength. Pos1 and Pos2 served as test points simulating a dual-user scenario, with the router transmitter operating at 5.2–5.8 GHz to fully cover the metasurface’s working band. “WiFi Magic Box 2024” intelligent detection software was employed to collect real-time parameters of received signals, including data rate and signal strength.

In the initial control experiment, Figure 5a shows the test environment without obstructions or a metasurface (router-to-phone distance~3 m), where Figure 5b indicates signal strengths of −32 dBm at both positions. When a transmissive metasurface was placed between the transmitter and receiver (Figure 5c), signal strengths at Pos1 and Pos2 increased to −26 dBm and −28 dBm, respectively—representing improvements of 6 dBm and 4 dBm over the initial state—due to the metasurface’s phase modulation. A further comparative experiment involved placing an obstruction of identical size to the metasurface in the same position (Figure 5e), reducing signal strengths to −36 dBm and −37 dBm. Results from multiple control experiments confirm that the metasurface effectively enhances signal strength at predefined positions via EM focusing, validating its practical utility in wireless communication scenarios.

## 3. Conclusions

This study presents a single-layer transmissive metasurface tailored for multi-user WiFi signal enhancement, leveraging geometric phase optimization and broadband unit cell design to address key challenges in indoor wireless communication. The symmetric unit cell structure with metallized vias forms a three-dimensional resonant mechanism, enabling effective phase coverage and high transmission efficiency, which outperforms conventional single-layer designs in balancing performance. Validated by simulations and measurements in both microwave anechoic chambers and practical office environments, the metasurface achieves stable multi-position focusing and creates simultaneous signal enhancement zones, effectively mitigating multipath fading and co-channel interference. Fabricated via mature PCB technology, this lightweight passive design avoids the complexity of multi-layer or active systems, facilitating low-cost deployment in indoor distributed networks and IoT scenarios. Future work will focus on extending operating bandwidth and exploring dynamic tunability while preserving efficiency, highlighting the potential of such single-layer metasurfaces as a scalable solution for optimizing multi-user wireless communication, bridging fundamental electromagnetic manipulation and real-world application needs.

## Figures and Tables

**Table 1:** Comparison of transmission achromatic metasurfaces.

	Unit Structure Layers	WiFi—Compatible	Average Efficiency
Ref. [46]	Multi-layers	N	Above 70%
Ref. [47]	One layer	N	54.6%
Ref. [48]	Multi-layers	N	75.7%
Ref. [49]	Multi-layers	N	78.9%
Our work	One layer	Y	81%

## Data Availability

The data that support the findings of this study are available from the corresponding author upon reasonable request.

## Supplementary Materials

The following supporting information can be downloaded at: https://www.mdpi.com/article/10.3390/mi16080911/s1, Figure S1. The S-parameters of two unit cells. Figure S2. The S-Parameters in different incident angles. Figure S3. The S-Parameters in different incident angles. Supplementary Information S1: Analysis of the electromagnetic characteristics of the structure under different thicknesses. Supplementary Information S2: Analysis of the electromagnetic properties of the structure under different thicknesses. Supplementary Information S3: Analysis of the electromagnetic characteristics of the structure under flexible substrates
