# Supplementary material for: Single-Layer High-Efficiency Metasurface for Multi-User Signal Enhancement
Source: Micromachines (Basel). 2025 Aug 6;16(8):911. doi: 10.3390/mi16080911 (PMC12388600; doi:10.3390/mi16080911)
Supplement: Supplementary file 1 [file micromachines-16-00911-s001.zip › micromachines-3794308-supplementary.pdf]

Supplementary Information:

Single-Layer High-Efficiency Metasurface for Multi-User Signal Enhancement

Hui Jin [1,2,3], Peixuan Zhu [1,2,3], Rongrong Zhu [4,*], Bo Yang [5], Siqi Zhang [1,2,3] and Huan Lu [1,2,3,*]

1 State Key Laboratory of Extreme Photonics and Instrumentation, ZJU-Hangzhou Global Scientific and Technological Innovation Center, Zhejiang University, Hangzhou 310027, China; 13706818052@139.com (H.J.); peixuanzhu@zju.edu.cn (P.Z.); sjgqzhang@outlook.com (S.Z.)
2 Zhejiang Key Laboratory of Intelligent Electromagnetic Control and Advanced Electronic Integration, Jinhua Institute of Zhejiang University, Zhejiang University, Jinhua 321099, China
3 International Joint Innovation Center, The Electromagnetics Academy at Zhejiang University, Zhejiang University, Haining 314400, China
4 School of Information and Electrical Engineering, Hangzhou City University, Hangzhou 310015, China
5 Sussex Artificial Intelligence Institute, Zhejiang Gongshang University, Hangzhou 310018, China; 18867645958@163.com
* Correspondence: rorozhu@zju.edu.cn (R.Z.); luhuan123@zju.edu.cn (H.L.)

Supplementary Information S1: Analysis of the electromagnetic characteristics of the structure under different thicknesses

We have systematically analyzed the electromagnetic responses under different substrate thicknesses (1mm, 3mm, 5mm, 7mm) through simulations, taking the typical unit cells numbered 4 and 5 in Figure 2c of the main text as examples. The results are shown in Figure S1 (Figures S1a and S1b present the transmission phase and amplitude of unit cell 4, while Figures S1c and R1d show the corresponding results for unit cell 5).

In terms of phase characteristics, within the 5.2-5.6GHz frequency band, the transmission phases of both unit cells exhibit regular shifts with changes in thickness, and the shift amount has a significant monotonic correlation with thickness (the phase shifts by an average of approximately 20° for every 2mm reduction in thickness). This regularity indicates that if an ultra-thin dielectric (such as 1mm) is adopted, the shift can be corrected by presetting a phase compensation amount, ensuring that the wavefront manipulation accuracy remains unaffected.

Regarding transmittance, the results show that it does not simply increase with decreasing thickness, but depends on the synergistic effect of dielectric thickness, relative permittivity (ε_r=2.65), and unit cell patterns. For unit cell 4, the transmittance is the lowest when the thickness is 1mm, which is because the excessively thin substrate leads to weak electromagnetic coupling between the metal pattern and the vias, thereby increasing radiation loss. In contrast, due to the difference in pattern structure, unit cell 5 achieves the highest

transmittance at 1mm, reflecting the key influence of the pattern on the energy transmission path.

In summary, although ultra-thin dielectrics (such as 1mm) can improve light transmittance, it is necessary to match the thickness according to the specific unit cell pattern design. For some unit cells (e.g., unit cell 5), lightweight design can be achieved while ensuring high transmittance; for others (e.g., unit cell 4), it is necessary to balance thickness and coupling strength. Therefore, in practical applications, if both high light transmittance and electromagnetic performance need to be considered, optimization can be achieved by selecting suitable combinations of unit cell patterns and thicknesses, and it is not necessary to blindly pursue ultra-thin designs in all scenarios.

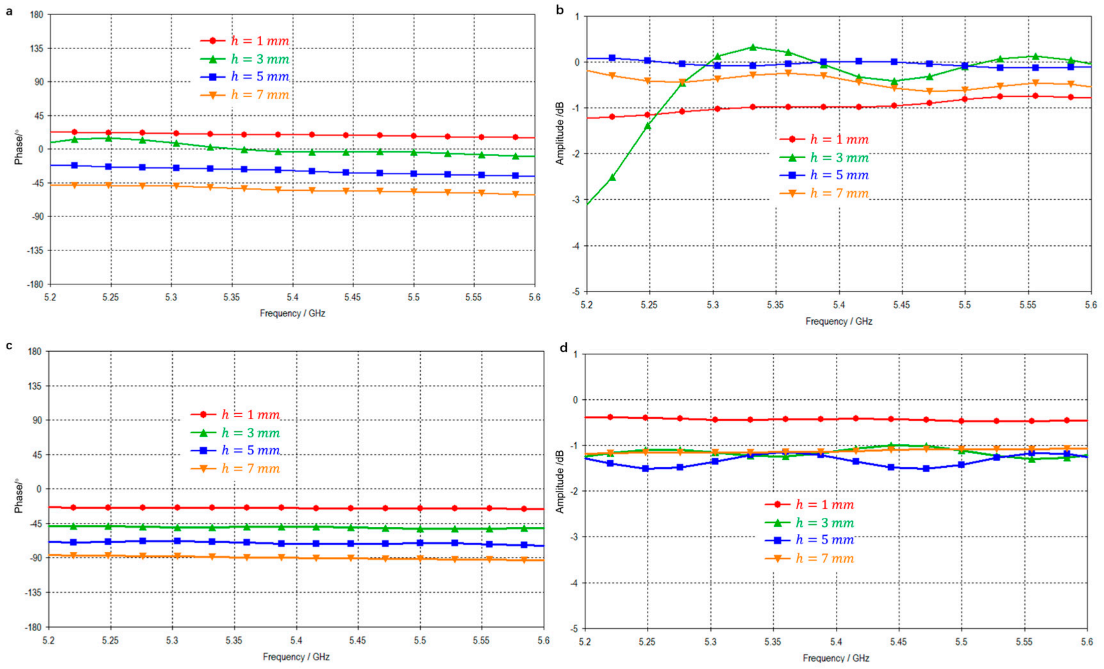

Figure S1. The S-parameters of two unit cells.

Supplementary Information S2: Analysis of the electromagnetic properties of the structure under different thicknesses

Addressing the characteristics of non-plane waves emitted by APs in indoor environments, we conducted oblique incidence simulation analysis using the unit structure numbered 4 in Figure 2c of the main text as an example. Within the incident angle range of 0-40°, the transmittance of this unit remains almost unchanged (with a fluctuation of <1%), and the phase response error can be controlled within approximately 7°, demonstrating excellent angular stability (as shown in Figure S2). Additionally, due to the symmetric structural design of the unit, its electromagnetic response remains consistent under different polarization modes, exhibiting good polarization stability. This indicates that even when faced with non-plane waves formed by reflection and scattering in complex indoor environments, the metasurface can still achieve

reliable WiFi signal enhancement through its stable angular and polarization characteristics.

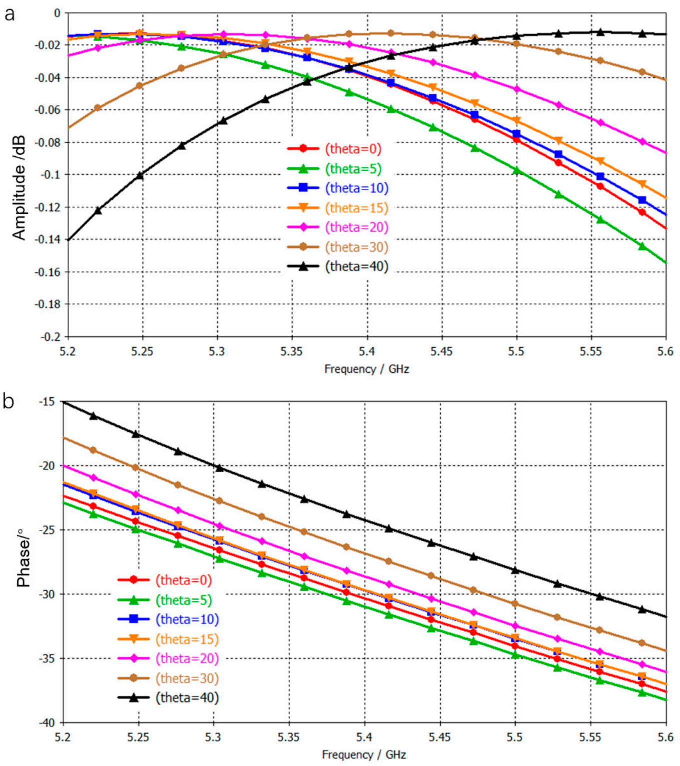

Figure S2. The S-Parameters in different incident angles.

Supplementary Information S3: Analysis of the electromagnetic characteristics of the structure under flexible substrates

We acknowledge the limitations of the rigid F4B substrate in curved and wearable applications. To address this, we have verified the application potential of flexible substrates through simulations: after replacing F4B with polyimide or PET (with a relative permittivity ε_r=3), the phase coverage of the unit in the 5.2-5.6 GHz frequency band can still reach over 270%, and although the transmittance decreases to some extent, it is generally controllable. This indicates that the structure is compatible with flexible carriers and can meet the requirements of conformal deployment. The simulation results using flexible substrates are shown in Figure S3.

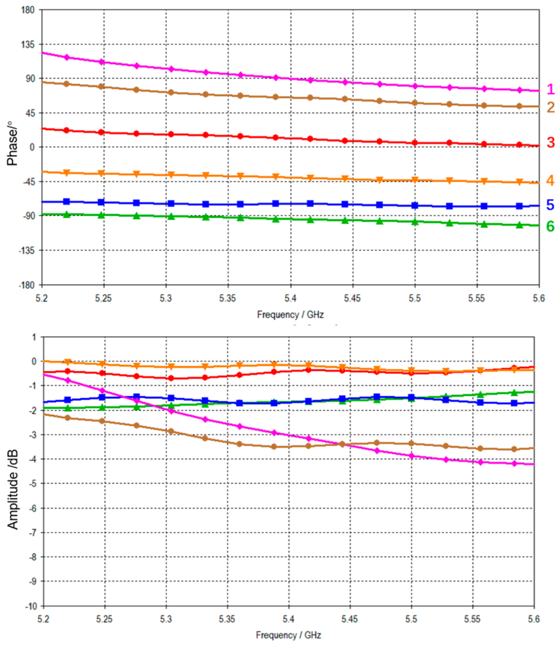

Figure S3. The S21 parameters.
